# Polypropylene Nanocomposites Attained by In Situ Polymerization Using SBA-15 Particles as Support for Metallocene Catalysts: Effect of Molecular Weight and Tacticity on Crystalline Details, Phase Transitions and Rheological Behavior

**DOI:** 10.3390/molecules28114261

**Published:** 2023-05-23

**Authors:** Rosa Barranco-García, Alberto García-Peñas, Enrique Blázquez-Blázquez, Jorge A. Ressia, Lidia M. Quinzani, Enrique M. Vallés, José M. Gómez-Elvira, Ernesto Pérez, María L. Cerrada

**Affiliations:** 1 Instituto de Ciencia y Tecnología de Polímeros (ICTP-CSIC), Juan de la Cierva 3, 28006 Madrid, Spain; rbarranco@ictp.csic.es (R.B.-G.); enrique.blazquez@ictp.csic.es (E.B.-B.); elvira@ictp.csic.es (J.M.G.-E.); ernestop@ictp.csic.es (E.P.); 2 Facultad de Ciencias Químicas, Universidad Complutense de Madrid, Avenida Complutense s/n, Ciudad Universitaria, 28040 Madrid, Spain; 3 Departamento de Ciencia e Ingeniería de Materiales e Ingeniería Química, IAAB, Universidad Carlos III de Madrid, Avda. de la Universidad, 30, 28911 Leganés, Spain; albertga@ing.uc3m.es; 4 Planta Piloto de Ingeniería Química (PLAPIQUI), UNS-CONICET, Camino La Carrindanga km. 7, Bahía Blanca 8000, Argentina; jressia@plapiqui.edu.ar (J.A.R.); lquinzani@plapiqui.edu.ar (L.M.Q.); valles@plapiqui.edu.ar (E.M.V.); 5 Comisión de Investigaciones Científicas de la Provincia de Buenos Aires (CIC), La Plata 1900, Argentina

**Keywords:** polypropylene, SBA-15 silica, in situ polymerization, hybrid particles, metallocene catalysts, nanocomposites, crystallites, confinement, rheological percolation

## Abstract

In this study, nanocomposites based on polypropylene are synthesized by the in situ polymerization of propene in the presence of mesoporous SBA-15 silica, which acts as a carrier of the catalytic system (zirconocene as catalyst and methylaluminoxane as cocatalyst). The protocol for the immobilization and attainment of hybrid SBA-15 particles involves a pre-stage of contact between the catalyst with cocatalyst before their final functionalization. Two zirconocene catalysts are tested in order to attain materials with different microstructural characteristics, molar masses and regioregularities of chains. Some polypropylene chains are able to be accommodated within the silica mesostructure of these composites. Thus, an endothermic event of small intensity appears during heating calorimetric experiments at approximately 105 °C. The existence of these polypropylene crystals, confined within the nanometric channels of silica, is corroborated by SAXS measurements obtained via the change in the intensity and position of the first-order diffraction of SBA-15. The incorporation of silica also has a very significant effect on the rheological response of the resultant materials, leading to important variations in various magnitudes, such as the shear storage modulus, viscosity and δ angle, when a comparison is established with the corresponding neat iPP matrices. Rheological percolation is reached, thus demonstrating the role of SBA-15 particles as filler, in addition to the supporting role that they exert during the polymerizations.

## 1. Introduction

Polyolefins, mainly polyethylene (PE) and polypropylene (PP), are by far the largest type of synthetic polymers globally used [1,2,3,4,5], constituting more than half of commercial plastic products. Their worldwide importance is owing to their low production costs, light weight, good processability and high chemical resistance. They also exhibit an incredibly broad range of mechanical properties, from elastomeric to thermoplastic behavior, and have fibers of a very high strength and modulus [6,7,8,9,10,11]. Thus, their overall market value has not stopped growing since they were first synthesized in the 1930’s, owing to their applicative potential in practically all production sectors, either as commodity products or in very specialized market domains. The combination of all these factors (that made possible their great success) is the reason for the current interest in polyolefins, which keep on developing with an anticipated increase in global demand of, on average, approximately 4.5% every year for the period 2014–2024. Their manufacture is, however, mainly derived from non-renewable resources and, in addition, polyolefins are neither compostable nor biodegradable. Thus, the very important challenges to be currently solved are, on one hand, the promotion of their massive reutilization through, for instance, mechanical recycling and, on the other hand, the development of an efficient chemical depolymerization method to be applied at the end of their life cycle in order to transform waste polyolefins into monomers or other byproducts. Both types of recycling would avoid waste accumulation in the environment, and would generate important economic benefits. Thus, further and future investigations are mandatory.

Important developments in reactor engineering and catalysis have contributed for many years to improving polyolefins’ synthetic process and final performance. Since the mid-1990s, metallocene catalysts have constituted a great tool in the production of polyolefins. Their use has provided remarkable versatility because numerous classes of polyolefins (homo- and copolymers by polymerizing cyclo-olefin or dienes, for instance) have become accessible [12,13,14], having previously been unattainable by any other catalytic systems or polymerization processes. Regarding PP, the use of these catalytic systems allowed the synthesis, under homogeneous conditions, of PP with low amounts of oligomers and different tacticities [15,16] (atactic, isotactic or isoblock, among others), and also copolymers with ethylene, 1-butene and longer chain α-olefins [17,18,19,20,21,22,23] with high comonomeric molar fractions. These synthetic developments enabled the tuning of properties depending on the ultimate requirements.

An important shortcoming of metallocene complexes at the industrial level is that they do not work successfully as homogeneous catalysts in the solution process. Thus, their immobilization is necessary in order to be employed in slurry and gas-phase reactors, such as the Ziegler–Natta catalytic systems. In supported catalysts for olefin polymerization, the support fragmentation process is crucial for preserving a high catalyst activity, managing the resultant morphology of the polymer particles, and attaining a uniform distribution of catalyst residues all over the polymeric matrix [24]. Silica and alumina were commonly used as inorganic supports [25,26,27] in early efforts to immobilize metallocene catalysts. Afterwards, zeolites [28] were also employed and showed interesting possibilities. Carbon nanomaterials (nanotubes, graphite, graphene oxide, among others) were also tried as carriers of either Ziegler–Natta or metallocene catalysts [29,30,31,32] in order to attain polypropylene-based materials via in situ polymerization. Current strategies are focused on the attainment of new supports or changes in the techniques of impregnation [33,34]. Mesoporous silicas, mainly MCM-41 [35] or SBA-15 [36], have received great attention regarding their capacity to support metallocene catalytic systems. These particles are constituted by an ordered structure of pores arranged in hexagonal frames, whose diameters range from approximately 2 to 20 nm. Both the confined polymerization of PE [37,38,39], as well as the production of PE nanofibers with metallocene Cp_2_ZrCl_2_ fixed on MCM-41 and SBA-15, have been demonstrated to take place [40,41]. Thus, they can be used as nanoreactors in polymerization. Self-reinforced nanocomposites have also been prepared, showing an improved mechanical performance and easier degradability, among others [38,42,43], which is very interesting in terms of sustainability. Efforts to enhance MCM-41dispersion (using either micro or nanosized particles) during in situ polymerization within the high-density polyethylene (HDPE) matrix have also been described [44,45,46]. Nanocomposites based on ultra-high-molecular-weight polyethylene (UHMWPE) and SBA-15 particles [47], and in-reactor blends of UHMWPE/HDPE [48], have also been reported.

In situ polymerization has also been found in the literature regarding the application of propylene with mesoporous silica using metallocene catalysts. They are, however, scarcer. A strategy involving the pretreatment of zirconocene with methylaluminoxane (MAO) before catalyst impregnation lead to an increase in the molecular weight [49]. Other studies have shown that the amount of catalyst immobilized grows when pretreating the support with MAO [50]. Improvements in catalytic activity were also achieved by substituting micro-sized MCM-41 for MCM-41 nanoparticles [51], owing to a reduction in the limitations on mass transfer. Composites of isotactic PP (iPP) and MCM-41 nanoparticles attained via in situ polymerization were also found in the literature [52], although their properties were not described.

The present work aims to obtain nanocomposites based on propylene by means of in situ polymerization using two different metallocene catalysts (the *rac*-ethylenebis(indenyl)zirconium (IV) dichloride (Et[Ind]_2_ZrCl_2_) and the *rac*-dimethyl-silylbis(1-indenyl) zirconium dichloride (Me_2_Si[Ind]_2_ZrCl_2_)), using the modified methylaluminoxane (MMAO) as a cocatalyst and using the mesoporous SBA-15 silica in a dual role: as catalyst support and as filler. As an early stage previous to the polymerization with propylene, hybrid SBA-15 particles, using these two metallocene catalysts, were prepared following an immobilization approach involving pre-activation (PA). This method implies the impregnation of these metallocene catalysts with MAO prior to its contact with SBA-15, which is the catalyst carrier. This route has been already applied to metallocene catalysts, being reported that the number of active sites increased and highly active catalysts were achieved [47]. This method also has the benefit of simplifying the experimental set-up during the immobilization stage. Once the catalyst is consumed during the synthesis, the SBA-15 silica is left in the final material in order to be additionally used as reinforcement. These two catalysts were selected because the molecular weight was supposed to be rather different either in the resulting homopolymers [53] or in the composites, this fact playing an important impact on the rheological response, which will be evaluated together with their thermal stability, crystalline characteristics and phase transitions. The resultant materials can be used in analogous applications other than iPPs, supposedly with an improved mechanical response. However, in addition, fine-tuning the protocols of the mesoporous silicas so that they act as catalyst supports could also allow the incorporation of other additional functionalities and enable them to play a key role during the use of the material, such as the addition of silver to provide antimicrobial characteristics [54], or to boost their decomposition at the end of its shelf life.

## 2. Results and Discussion

### 2.1. Polymerizations and Microstructure of the Obtained Nanocomposites

The average activities of the different polymerizations were estimated using the total amount of sample obtained. The reaction conditions remained unchanged, independent of the metallocene catalyst used; this is except for the reaction time, which was the only parameter driving the global activity found and, thus, the content of SBA-15 in the final composite [53]. The average activity of the polymerizations performed using the Et[Ind]_2_ZrCl_2_ catalyst was found to decrease with the reaction time, as listed in Table 1. This behavior is ascribed to several factors, among which the deactivation of the catalyst and the hindrance of monomer diffusion through the polymer, which covers the hybrid catalyst-supported SBA-15 particles, play the most important roles [47,48]. It can also be deduced from Table 1 that the high average activity observed for the polymerization carried out under homogeneous conditions with Et[Ind]_2_ZrCl_2_ is preserved in the presence of SBA-15 at low reaction times.

The average activities of the materials prepared using the Me_2_Si[Ind]_2_ZrCl_2_ catalyst are much lower than those just mentioned, as seen in Table 1. Nevertheless, a similar qualitative trend is exhibited, i.e., a decrease in activity with the reaction time. Then, the highest values are noticed at the short reaction times, even in the presence of SBA-15. The significant decrease that occurs in the materials prepared using this Me_2_Si[Ind]_2_ZrCl_2_ catalyst is ascribed to the lower insertion rate of propylene, as well as to the higher sensitivity that this Me_2_Si[Ind]_2_ZrCl_2_ catalyst shows against the presence of impurities during the reaction process, compared with that exhibited by Et[Ind]_2_ZrCl_2_ [53]. Therefore, their deactivation processes are maximized to a great extent. Similar low values have been described in polypropylene systems in which the Me_2_Si[Ind]_2_ZrCl_2_ catalyst is supported onto mesoporous MCM-41 silica [52].

Another parameter that is collected in Table 1 is the molar mass (M_w_). The use of the Me_2_Si[Ind]_2_ZrCl_2_ catalyst leads to longer polypropylene chains, i.e., the values of molar masses shown are higher than those achieved when the Et[Ind]_2_ZrCl_2_ catalyst is used [53]. The differences in the molecular weights are rather significant. A further increase in this molecular parameter is noticeable in the materials containing the SBA-15 particles, i.e., where the catalytic system has been supported on the mesoporous silica, independent of the catalyst used. This enlargement of molar masses in the nanocomposites has been previously described when using a nitrogen-flushed bottle as a reactor in pressure reactions [55]. The rise is more considerable when the Me_2_Si[Ind]_2_ZrCl_2_ catalyst is employed compared to that noted in the systems prepared by means of the Et[Ind]_2_ZrCl_2_ one.

The tacticity distribution was also analyzed since it is another very remarkable molecular parameter in iPP-based materials. These results are listed in Table 2, including the relative content of the different triads, as measured by ^13^CNMR, together with the content of carbons involved in regio-defects. It seems clear from these results that the catalyst Me_2_Si[Ind]_2_ZrCl_2_ leads to materials with more stereo and regio-regular PP macrochains. It is also noticeable that the presence of SBA-15 improves the isotacticity of both catalysts and the regio-regularity of the PP samples obtained using Et[Ind]_2_ZrCl_2_.

Improvements in the regularity and chain size are undoubtedly associated with metallocene catalysts supported on Si-based particles [56,57], and are the result of a more sterically hindered propylene insertion and more constrained dynamics of the growing chains, respectively [58].

### 2.2. Thermal Stability and Morphological Characteristics

**Figure 1:**
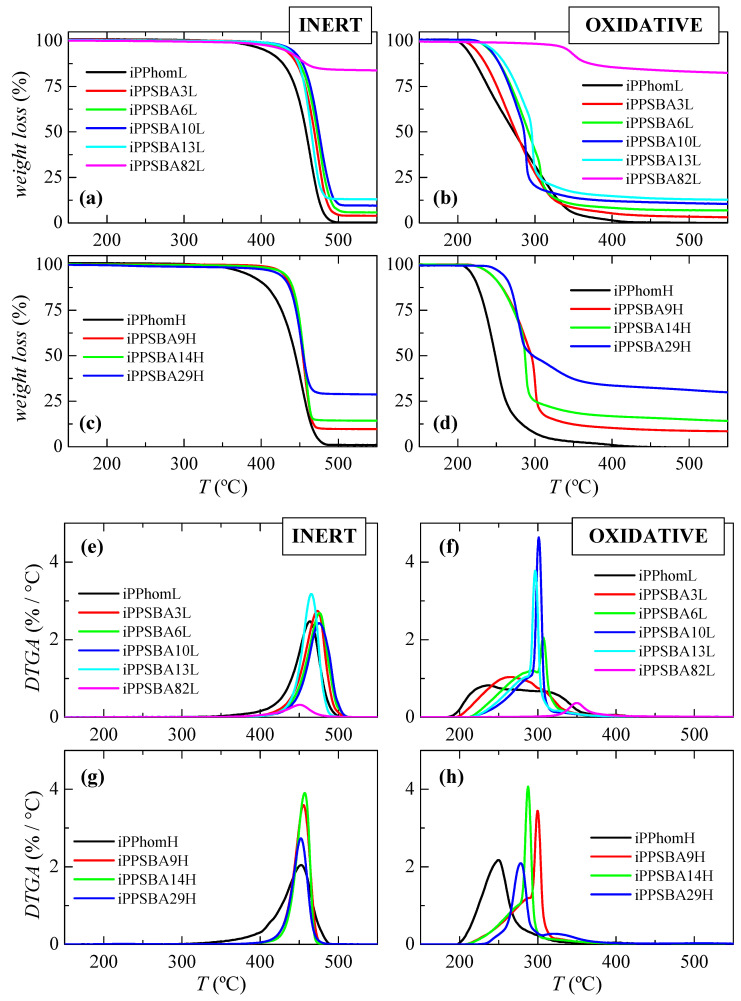
TGA curves under inert (**a**,**c**) and oxidative (**b**,**d**) atmospheres and their corresponding DTGA derivatives ((**e**,**g**) for an inert medium together with (**f**,**h**) for an oxidative environment) for the iPP homopolymers and their composites using SBA-15 at different contents prepared via in situ polymerization: materials synthesized using the Et[Ind]_2_ZrCl_2_ catalyst in the top representations, and those attained by means of the Me_2_Si[Ind]_2_ZrCl_2_ catalyst in the bottom representations.

Figure 1a–d represents the TGA curves used to analyze the thermal stabilities of the iPP homopolymers (iPPhomL and iPPhomH) and those of the different nanocomposites obtained by using the hybrid SBA-15 particles and these two catalytic systems. The thermal degradation was analyzed under two environments: inert, employing pure nitrogen (N_2_), and oxidative, through the use of air. Figure 1a–d shows that both the anaerobic and oxidative degradations of the iPP samples are delayed in the presence of SBA-15, this stabilization being evident even at the lowest SBA-15 contents. This delay is observed to a lesser extent at higher SBA-15 contents. Such trends are qualitatively similar in the two series of composites obtained using both catalysts. However, the molar mass seems to play a role, since the comparison of the nanocomposites with the analogue SBA-15 content shows some differences. The temperature at which the material starts to lose weight (T_i_) under inert conditions is 455.0 °C for iPPSBA10L, while it appears at 440.5 °C in iPPSBA9H; in addition, this value is 449.5 °C for iPPSBA13L, while it is 443.0 °C for iPPSBA14H. This means that long iPP macrochains, i.e., those with higher molar masses, initiate their decomposition in the presence of SBA-15 particles at lower temperatures than the ones with shorter lengths. The influence of the molar mass on T_i_ is less significant when the experiment is performed under an air environment, with T_i_ values of 255.5, 251.0, 253.5 and 246.5 °C for iPPSBA10L, iPPSBA13L, iPPSBA9H and iPPSBA14H, respectively, being obtained.

The behavior of these composites showing increasing degradation temperatures agrees well with that described for the iPP/SBA-15 nanocomposites prepared using melt extrusion [59]. The incorporation of SBA-15 particles exerts a positive impact on the iPP stability, independent of the atmosphere of the experiment and despite the existing differences between the mechanisms involved in the thermal degradation of iPP when the process takes place under an inert or air environment. In both cases, the presence of the SBA-15 silica allows the viscosity of the melt to rise in the different composites, as will be discussed later on. This increase in viscosity constrains chain dynamics, which has two main effects; on the one hand, the possibility of bond breakage is reduced when experiments are carried out under an N_2_ atmosphere, and, on the other hand, the diffusion of air into the material bulk is hindered when measurements take place under oxidative conditions.

Different trends regarding the initial temperature values (T_i_) with molar mass are observed in the iPP homopolymer samples when the results obtained under the inert or the oxidative environments are compared. In the former, T_i_ is 437.5 °C for iPPhomL and 420.5 °C for iPPhomH; meanwhile, the values are 212.0 °C for iPPhomL and 225.5 °C for iPPhomH in experiments conducted run under air. More specific interactions in the molten state (intrachain conformational distortions or entanglements, among others) have been found to take place in the chains with a higher molar mass. These interactions behave as weaker points under an inert atmosphere at high thermal stresses and, thus, stability seems to be reduced in specimens with higher molecular weights [60].

On the other hand, it is well known that the oxidative degradation of iPP is oxygen-diffusion-controlled [61], and it is clear that the ability of oxygen to enter into the molten bulk is thus greater in the samples with a low molecular weight, with their degradation being expected at lower temperatures. This anticipated dependence on molar mass observed in the neat homopolymer is not noticed in the composites at similar silica contents. Thus, it seems that specimens with a lower molecular weight exhibit the highest T_i_ values, independent of the experimental environment and the content of the mesoporous SBA-15. As aforementioned, oxidant media are more aggressive to shorter macrochains, but the SBA-15 that is present seems to interact with the oxygen in the air, leading to a more controlled release that provides protection to those chains with a lower molecular weight. Differences between the T_i_ are reduced when a comparison is established in this more hostile environment.

In order to obtain more information, the derivatives of the TGA curves are represented in Figure 1e–h. Degradation under nitrogen clearly takes place via a unique process, independent of the molar mass of the homopolymers and composites, as already deduced from Figure 1a,c. Quite similar curves in terms of their position and shape are noticeable in all of the samples containing the SBA-15 silica. Instead, two clear maxima are observed in these derivative curves for the experiments performed in an oxidative environment: a broad process at a low temperature, together with a much narrower one above it. A rising resistance against oxidation is noted in the composites. The presence of the SBA-15 particles might force the diffusion of air through distorted pathways within the molten PP matrix, hindering its decomposition [61]. The shape found in the DTGA of the iPPhomL homopolymer seems to point out the microstructural heterogeneity present in this sample, which suggests that its degradation occurs along a broad temperature interval.

As aforementioned, SBA-15 particles were employed as carriers of the catalysts during the polymerization and they were not eliminated when the synthetic process was finished, meaning that silica can act additionally as filler. The estimation of the SBA-15 amount is then mandatory in order to quantify its real influence over properties. Thermogravimetric analysis is an appropriate and simple tool that can be used to determine the content of inorganic constituents, together with information about the thermal stability and degradability. Measurements were carried out in order to obtain reliable values under two different environments (inert and oxidative), as mentioned, from either the initial polymerization powders or from the films obtained after their compression molding. Then, for a given material, the average SBA-15 content, reported in Table 1 and Table 2, is the mean value derived from these four different measurements. Quite similar SBA-15 results were achieved from all of these experiments, indicating rather good homogeneity in the amount and distribution of the silica within the nanocomposites. This is connected with the morphological aspects mentioned below.

**Figure 2:**
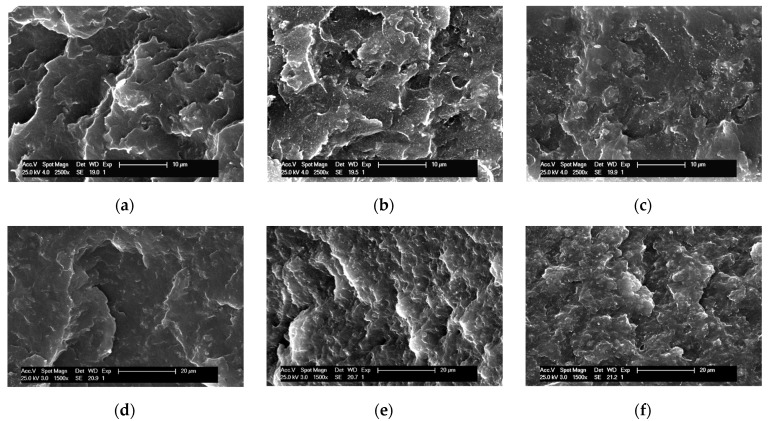
SEM micrographs of different nanocomposites obtained via in situ polymerizations using the Et[Ind]_2_ZrCl_2_ catalyst: (**a**) iPPSBA6L, (**b**) iPPSBA10L, (**c**) iPPSBA13L; and, employing the Me_2_Si[Ind]_2_ZrCl_2_ catalyst: (**d**) iPPSBA9H, (**e**) iPPSBA14H and (**f**) iPPSBA23H.

Figure 2 shows the morphological features found on the fracture surface of several composites prepared via in situ polymerization using both catalysts, Et[Ind]_2_ZrCl_2_ and Me_2_Si[Ind]_2_ZrCl_2_, and supported on the mesoporous SBA-15 particles. Similarities and differences are noticed when a comparison is established between the composites achieved using both catalytic systems. The main similarity is related to the dispersion of the silica particles, this being homogenous and uniform in these two families, independent of the resultant molar mass of the iPP matrix and of the content of silica. In situ polymerization has been described as a protocol by which to induce intimate matrix/filler contact, even in cases with high differences between the chemical characteristics of the constituents, such as here, where iPP is hydrophobic and SBA-15 is highly hydrophilic [43,45]. This closeness at the interface boosts filler dispersion and avoids the formation of particle agglomerates that are large in size.

On the other hand, the differences observed are ascribed to the morphology of the fracture surface associated with a higher fragility during the breaking protocol of the composites obtained using the Et[Ind]_2_ZrCl_2_ catalyst. The iPP within these composites reaches a much lower molecular weight than that in the materials prepared using Me_2_Si[Ind]_2_ZrCl_2_. Furthermore, the incorporation of a hard component (in the current case of SBA-15 silica) usually leads to a reinforcement effect that mostly increases the rigidity and decreases the deformability of the final material. These two factors, the low molar mass and presence of silica, increase the fragility of the composites prepared using the Et[Ind]_2_ZrCl_2_ catalyst, and the fracture surface becomes sharp, mainly at the lowest contents, as seen in the iPPSBA6L picture or when iPPSBA10L and iPPSBA9H are compared.

### 2.3. Phase Transitions and Crystalline Details

**Figure 3:**
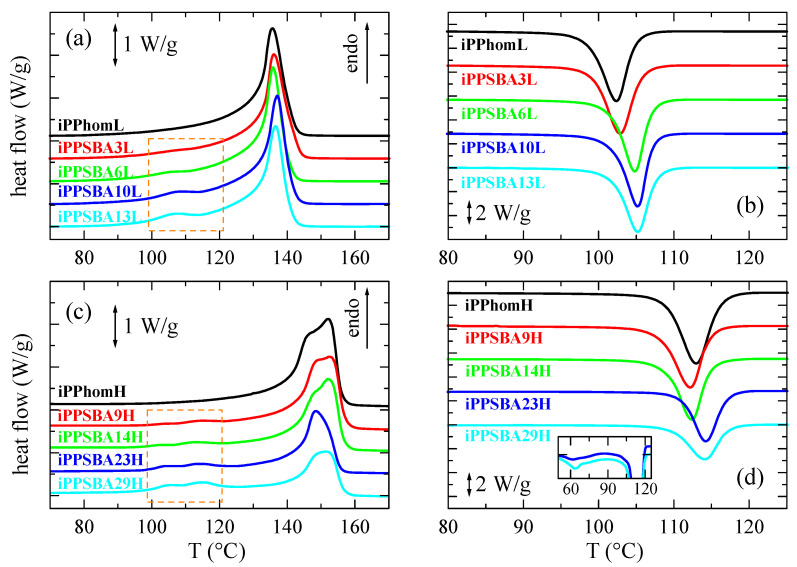
(**a**) DSC endotherms related to the first melting run, shifted along the *y* axis for a better visualization of the low-molecular-weight samples (**a**) and the high-molecular-weight samples (**c**); DSC exotherms attained during cooling of the melt for the low-molecular-weight samples (**b**) and the high-molecular-weight samples (**d**).

Figure 3 displays the calorimetric curves attained during the first melting and cooling processes for the different nanocomposites prepared using both catalytic systems. As already mentioned, the iPP of these two families, obtained via the in situ polymerization of propylene using the hybrid SBA-15@Et[Ind]_2_ZrCl_2_ or SBA-15@Me_2_Si[Ind]_2_ZrCl_2_ particles, respectively, shows differences in its molecular weight and tacticity, in such a way that the materials obtained using the former show lower molar masses and are less stereo-regular, as reported in Table 1 and Table 2. Relative to the heating runs, two distinct endothermic events are noticed in the represented temperature range: the main one takes place at approximately 136 °C or 150 °C for the sets prepared using the SBA-15@Et[Ind]_2_ZrCl_2_ or SBA-15@Me_2_Si[Ind]_2_ZrCl_2_ catalytic systems, respectively, and the other process, of a much smaller intensity, occurs at approximately 110 °C. The primary peak is associated with the melting of regular polypropylene chains, and the differences found in the melting temperature (T_m_) of these two families are due to the smaller crystallite size developed in the materials obtained using the hybrid SBA-15@Et[Ind]_2_ZrCl_2_ silica; this is owing to the inferior regularity of their macrochains, as estimated via NMR and listed in Table 2.

The process appearing at approximately 110 °C, which is highlighted in Figure 3, is ascribed to polypropylene crystallites that are capable of developing in the nanometric spaces that are within the SBA-15 mesostructure. The existing geometrical restrictions, because the diameter of the pores is approximately 6 nm [62], prevent the iPP chains on the inside of the channels from crystallizing as thick as those on the outside of the SBA-15 particles; consequently, the derived crystallites cannot grow more than the pore volume. Thus, the resultant crystal size is lower and their T_m_ is displaced to lower temperatures than those for the main melting process. Similar behavior has been observed in iPP materials prepared via extrusion [63]. Furthermore, the appearance of an endothermic event at lower temperatures than the primary endotherm has been described when using MCM-41 instead of the SBA-15 silica in HDPE nanocomposites prepared via in situ polymerization [38,44,46], as well as nanocomposites of iPP [64] or polycaprolactone (PCL) obtained via extrusion [65]. In these materials containing MCM-41 particles, the T_m_ of the confined crystallites is, however, shifted down compared to the ones obtained using the SBA-15 silica because the pores in MCM-41 are smaller than in SBA-15 [35,36].

It can be also deduced from Figure 3 that the total enthalpy involved in the main melting process displays a decreasing trend as the SBA-15 content increases in the materials synthesized using SBA-15@Me_2_Si[Ind]_2_ZrCl_2_; in addition, there is almost constancy in the ones polymerized in the presence of SBA-15@Et[Ind]_2_ZrCl_2_. The intensity of a phase transition, such as melting, depends on the iPP amount because the event is related to the polymeric matrix. Thus, enthalpy values require normalization to the actual content of iPP in a given composite. Two different trends are noted depending on the catalytic system used. In materials with the smallest molar mass and a less regular iPP, the presence of SBA-15 leads to an increase in enthalpy (and crystallinity) as the silica content rises, while similar or slightly reduced values are noticeable relative to the silica amount for the high-molecular-weight samples, as listed in Table 3. The behavior of the material family prepared using the hybrid SBA-15@Et[Ind]_2_ZrCl_2_ particles is somehow different to the one observed in the iPP nanocomposites obtained via extrusion [63], which is more analogous to the one found in the set polymerized using SBA-15@Me_2_Si[Ind]_2_ZrCl_2_. This dissimilarity is ascribed to differences in the molar mass.

Regarding the small endotherms, which arise from the iPP crystals confined within the SBA-15 pores, the enthalpy involved increases as the SBA-15 content is raised; this is because the volume inside the pores increases accordingly and more iPP chains can be accommodated inside the silica channels. A comparison between the normalized values found in iPPSBA10L and in iPPSBA9H, which are 3.5 and 2.5 J/g, respectively, indicates that an iPP of a lower molar mass crystallizes easily in the SBA-15 mesostructure. These values were obtained via the integration of heating curves for the different samples in the temperature interval range of 98 to 122 °C after baseline subtraction.

In relation to the T_m_ of the primary melting process, there is not a significant change in the location of T_m_ for a specific family, being in the composites very slightly higher than in the iPP homopolymer. The T_m_ values are, however, greater in the specimens prepared using the Me_2_Si[Ind]_2_ZrCl_2_ catalyst than in the samples using the Et[Ind]_2_ZrCl_2_ catalyst. The reason for those higher T_m_ values in the former is ascribed to the higher regularity of their iPP chains, as demonstrated by the NMR results (see Table 2). The melting process is bimodal in the samples prepared using the Me_2_Si[Ind]_2_ZrCl_2_ catalyst because the higher iPP molar mass promotes the melting–recrystallization–melting processes during the heating cycle.

Figure 3b,d display the crystallization exotherms for the different materials. Two different trends can be observed. The incorporation of SBA-15 particles into the composites containing the iPP of a low molar mass plays a nucleating effect, i.e., the crystallization temperature (T_c_) moves to a higher temperature compared with that exhibited by the iPP homopolymer. On the contrary, the T_c_ appears at a slightly lower temperature than for the iPP matrix at the smallest silica contents in the composites in which the molecular weight of iPP is much greater; however, the T_c_ values are higher than for the homopolymer at SBA-15 quantities above 20 wt.%. Thus, for the high-molecular-mass samples, the iPP crystallization is slightly hindered in the lower SBA contents and boosted for the higher silica contents. Furthermore, the crystallization of the confined iPP chains can be observed relative to cooling in the iPPSBA23H and iPPSBA29H specimens. It occurs at a lower temperature than the main crystallization, as expected, because these crystals are much smaller since they have to be developed in a nanometric space confined by the walls of the SBA-15 pores. Therefore, it usually takes a long time, especially in the cooling experiments [63], and it is only noticeable at relatively high quantities of silica (see inset in Figure 3d).

**Figure 4:**
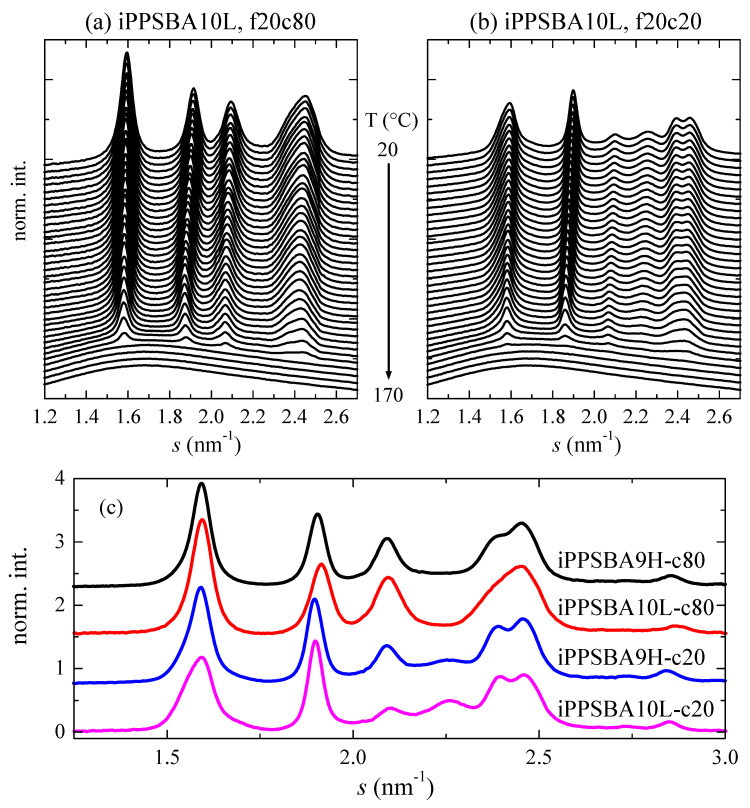
(**a**) Real-time variable-temperature profiles at a wide angle obtained using synchrotron radiation for the heating of the iPPSBA10L sample at 20 °C/min in the first melting process (f20c80) and (**b**) in its second melting after crystallization at 20 °C/min (f20c20). (**c**) Patterns for the initial rapidly cooled (at approximately 80 °C/min, c80) iPPSBA9H and iPPSBA10L specimens at room temperature, as well as after their crystallization at 20 °C/min (c20). For clarity, only one every two frames is plotted in (**a**,**b**).

Several crystalline polymorphs in iPP have been described, namely α, β, γ, δ, as well as a mesomorphic form, which can be tuned by variations in the temperature/pressure/cooling rate applied during processing, along with the addition of specific nucleating additives and changes in microstructural characteristics [21,23,66,67,68,69,70,71,72,73,74,75,76]. Figure 4 shows the real-time variable-temperature profiles at a wide angle (WAXS) obtained using synchrotron radiation during the first and second heating experiments for the iPPSBA10L sample.

The characteristic reflections associated with the monoclinic α lattice are noticeable in the patterns during the melting just after processing. The appearance of this crystalline cell is expected [77], since the film has been prepared via compression molding and the subsequent imposition of a relatively rapid cooling from the melt. No evidence of the formation of the γ modification is detected under these processing conditions, despite the fact that the catalyst used during its synthesis is a metallocene and the sample has a low molecular weight (see value in Table 1). This orthorhombic polymorph, easily identified because of its characteristic (117) reflection [78] at approximately 2.25 nm^−1^, is, however, noted in the heating profiles after a cooling at 20 °C/min (see in Figure 4b); this is because the γ modification is boosted at low crystallization rates [77,79].

On the other hand, the presence of the SBA-15 silica does not influence the crystalline lattice developed in the nanocomposites processed under these conditions, and exhibits all of the characteristic reflections of the monoclinic cell in the first melting and a certain amount of the two crystalline forms in the second heating process. These features are in good agreement with the crystalline features observed in the nanocomposites of iPP with SBA-15 prepared via extrusion [63]. A comparison of the iPPSBA10L and iPPSBA9H profiles at room temperature reveals that no differences can be observed during the first heating experiments, with only the monoclinic modification being developed. The crystallinity values are 0.62 and 0.55 for iPPSBA10L and iPPSBA9H, respectively, for this monoclinic form. Nevertheless, crystallization at 20 °C/min enables a higher amount of the orthorhombic polymorph to be obtained in iPPSBA10L than in iPPSBA9H, as deduced from the higher intensity of its (117) diffraction (at *s* position of approximately 2.25 nm^−1^). In fact, the total crystallinity is 0.56 and 0.54 for iPPSBA10L and iPPSBA9H, respectively, and the content in the orthorhombic lattice is 56% in the former, while it is only 16% in the iPPSBA9H composite. This lattice is promoted in iPP with a low molecular weight, and the iPPSBA10L sample, synthesized using the hybrid SBA-15@Et[Ind]_2_ZrCl_2_ support, shows a much reduced molar mass compared to the iPPSBA9H nanocomposite.

**Figure 5:**
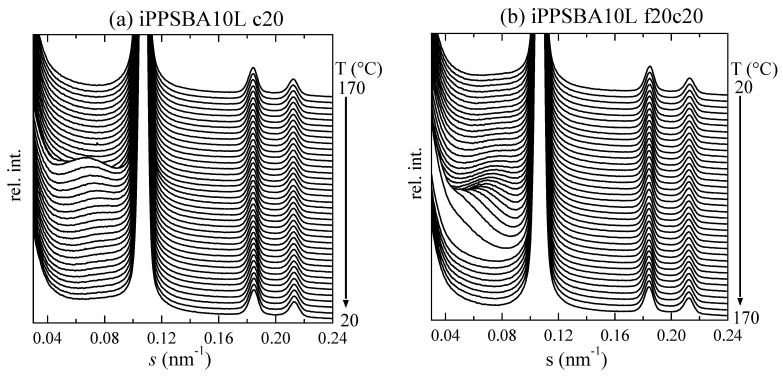
Real-time variable-temperature SAXS profiles obtained using synchrotron radiation for the iPPSBA10L sample corresponding to the cooling from the melt at 20 °C/min: (**a**) and subsequent melting at the same rate (**b**). Only one every two frames is plotted for clarity.

In order to obtain a deeper understanding of the influence of the incorporation of SBA-15 into the iPP crystalline structure, real-time variable-temperature experiments were also performed simultaneously at the small-angle region (SAXS). In this interval of the scattering vector, information is obtained on the long spacing of the iPP, as well as on the characteristic diffractions of the hexagonal morphology of SBA-15 in the nanocomposites; this is clearly seen in Figure 5, in which cooling at 20 °C/min and the subsequent heating experiments, also at 20 °C/min, are represented (left and right, respectively) for the iPPSBA10L composite. The iPP long spacing appears in the homopolymer and in the nanocomposites at *s* values smaller than the primary SBA-15 reflection. As the cooling experiment starts (see top profiles in Figure 5a), the iPP is in its amorphous state, i.e., a unique isotropic phase exists and no differences in the electron density are noticed. Consequently, long spacing is not observed because this is a parameter employed in the theory of the lamellar stack model for semicrystalline polymers, which are associated with differences in the electron density between the amorphous and crystalline phases. This electron density changes significantly during the crystallization, recrystallization or melting processes in semicrystalline polymers [80,81,82,83], becoming an important means for the characterization of all of these phase transitions. At around 105 °C/min, crystallization takes place and a broad peak appears; this is related to long spacing and is centered at approximately 0.06 nm^−1^. Its position is displaced to higher *s* values and its intensity is reduced as temperatures decrease. At room temperature (back profile), the iPP long spacing partially overlaps with the SBA-15 first-order diffraction.

The observations made during the subsequent heating are basically opposite: long spacing remains rather unchanged up to approximately 80 °C; then, a rise in the intensity and a displacement to inferior *s* values is noted, which become more important above 110 °C, where considerable crystal thickening occurs. Afterwards, its vanishing takes place at approximately 140 °C, which is related to the melting process of those iPP crystallites within the macrochains located outside the SBA-15 particles. This behavior is rather analogous for all of the specimens analyzed. The only difference is connected to the location of the several phase transitions; this is because, as aforementioned, the materials prepared using the hybrid SBA-15@Me_2_Si[Ind]_2_ZrCl_2_ particles develop a higher tacticity and greater chain regularity Consequently, they crystallize and melt at higher temperatures than the iPPhomL and its nanocomposites polymerized in the presence of the SBA-15@Et[Ind]_2_ZrCl_2_ silica.

On the other hand, the mesoporous SBA-15 silica shows an ordered array that is ascribed to its *p6mm* hexagonal symmetry [84]. In Figure 5, the (100) first-order reflection, which is located at an *s* position of 0.107 nm^−1^, is partially represented together with the (110) and (200) diffractions (whose locations in the *s* scale are at 0.185 and 0.213 nm^−1^, respectively) of its periodically organized two-dimensional structure in the iPPSBA10L nanocomposite. The (100) peak of this hexagonal arrangement is very intense and it is cut in order to be able to detect the iPP long-spacing peak and the other two (110) and (200) reflections, which are of a much smaller intensity. Observation of the characteristic SBA-15 diffractions proves that silica, after acting as a catalyst carrier, satisfactorily remains within the iPP matrix in all of the nanocomposites at the end of the in situ synthetic protocol, despite some iPP chains being produced within the their mesopores, as suggested by the small endothermic peak at around 110 °C in the DSC experiments (see Figure 3).

In relation to the existence of polymeric chains, in the case of those of the iPP, which are confined within the nanometric pores, a careful evaluation of the SAXS results using synchrotron radiation on the main (100) diffraction of SBA-15 reveals a key sensor of the presence of crystallites within the mesostructure. This has been demonstrated in iPP and PCL-based materials with SBA-15 prepared via extrusion [63,85,86]. It was shown that location of the (100) peak in the neat SBA-15 was practically unchanged (similar to its intensity and width, which were constant) in the temperature interval analyzed, from 20 to 200 °C. On the contrary, variations in the intensity of the SBA-15 peak were found in the iPP and PCL composites just mentioned [63,85,86] during the heating and, sometimes, cooling stages. It was associated with the two phase transitions, melting and crystallization, of the corresponding confined crystals developed using the iPP chains that grew during polymerization within the SBA-15 pores. These crystallites have to be adjusted to the nanometric diameter and thus their lateral size is very small. Consequently, the confined crystals melt at temperatures significantly lower than those for the crystallites outside the channels. The existence of polymeric chains in these nanometric spaces enables the polymerization occurring partially inside the SBA-15 pores to be assured and makes it possible to name these materials as nanocomposites. These findings agreed with earlier results obtained concerning other SBA-15 hybrids [87,88,89], where it was suggested that the intensity of the SBA-15 diffraction was dependent on the degree of pore filling and also on the scattering contrast that eventually exists between the walls and interior of the SBA-15 channels.

**Figure 6:**
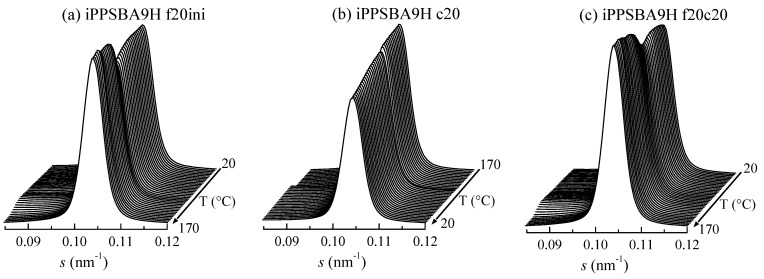
Synchrotron SAXS 1D diffractograms, in the region of the SBA-15 (100) peak, for the iPPSBA9H sample: (**a**) first melting, (**b**) crystallization and (**c**) subsequent melting processes, all at a scanning rate of 20 °C/min. For clarity, only one every two frames is plotted.

Figure 6 shows the real-time variable-temperature profiles during the first heating, cooling and subsequent heating for the iPPSBA9H nanocomposite in the region of the SBA-15 (100) peak. In the heating runs, a clear discontinuity in the (100) intensity is noted in such a way that at room temperature, its height is smaller than at 170 °C. The electronic density of the iPP chains within the SBA-15 pores is different when they are in a semicrystalline state, comprising two phases (amorphous and crystalline), than when they are constituted by a single amorphous phase; in addition, the change takes place during the melting of these confined crystallites, at around 110 °C in the first heating and at lower temperatures (around 103 °C) for the heating after crystallization at 20 °C/min. A progressive variation in intensity in the (100) reflection is, however, noticeable relative to the cooling, as depicted in Figure 6b.

**Figure 7:**
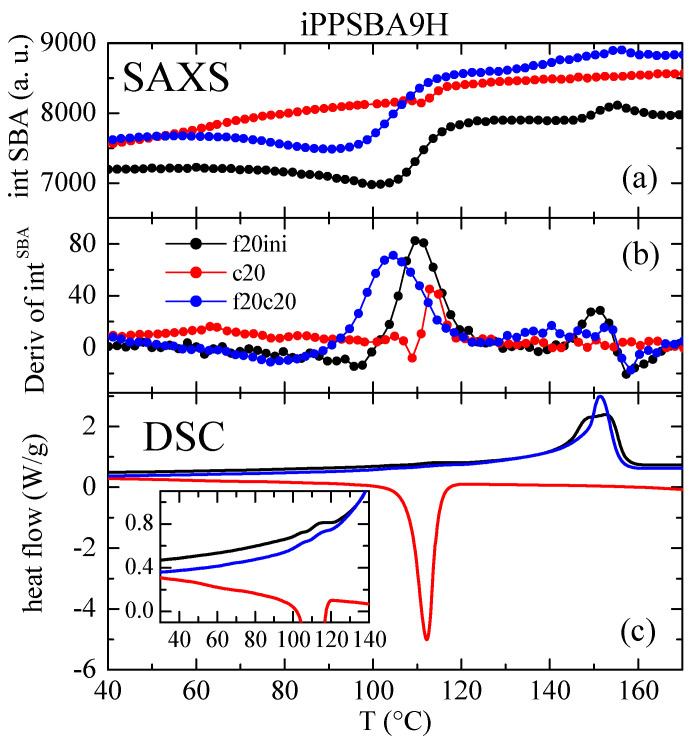
(**a**) Variation of intensity with temperature for the SBA-15 (100) diffraction in the iPPSBA9H nanocomposite relative to the initial heating (f20ini), cooling (c20) and subsequent heating (f20c20), with all of them scanning at 20 °C/min. (**b**) Corresponding derivative of that variation, compared with their analogous DSC curves (**c**). Black color stands for f20ini, red for c20 and blue for f20c20 in the different plots.

**Figure 8:**
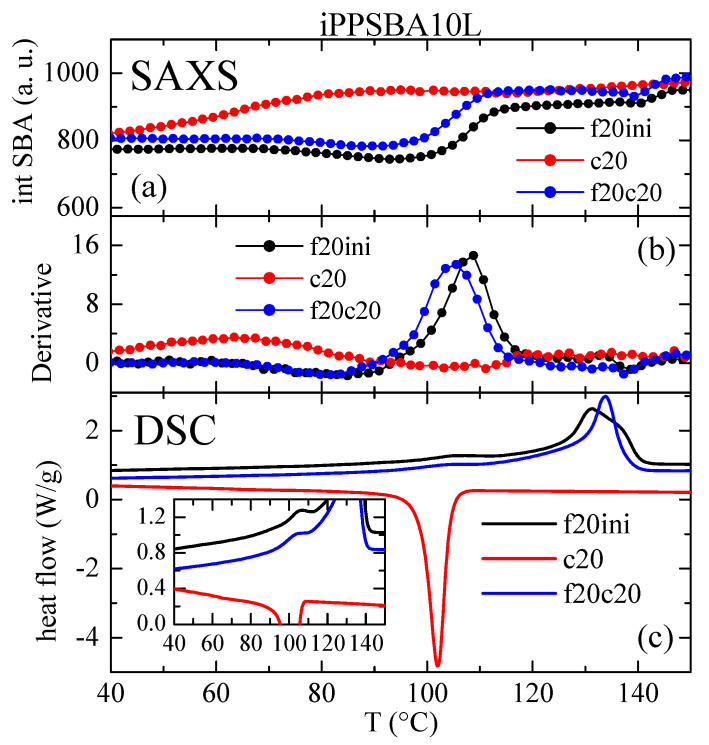
(**a**) Variation of intensity with temperature for the SBA-15 (100) diffraction in the iPPSBA10L nanocomposite relative to the initial heating (f20ini), cooling (c20) and subsequent heating (f20c20), with all of them scanning at 20 °C/min. (**b**) Corresponding derivative of that variation, compared with their analogous DSC curves (**c**).

These changes in intensity for the principal (100) diffraction of the SBA-15 particles can be quantified as a function of temperature, as represented in Figure 7 and Figure 8 for the iPPSBA9H and iPPSBA10L nanocomposites, respectively; these act as examples for each of the two families synthesized. Thus, the variation in the (100) intensity and its derivative are plotted in these two Figures, and are compared with the DSC curves for the first heating, crystallization and subsequent heating cycles. A very good agreement is seen between the results provided by these two techniques.

Thus, the peak area rises clearly and systematically in all of the iPP nanocomposites in the range of approximately 90 to 120 °C, as seen in Figure 7 and Figure 8 for the iPPSBA9H and iPPSBA10L, respectively. In fact, the maximum in the derivative appears at 113.5 and 108 °C for iPPSBA9H and iPPSBA10L; meanwhile, from the DSC heating curve, the maximum in the derivative was observed for these two samples at 115 and 108.5 °C, respectively. Thus, the interpretation of the DSC results depicted in Figure 3a,c, is now fully corroborated by SAXS measurements. The occurrence of confinement in some iPP macrochains is characterized by endothermic events taking place at a considerably lower melting temperature; this is due to the small size of these ordered entities, as mentioned above. The simultaneous variation in the SAXS intensity for the main SBA-15 diffraction during heating is, then, attributed to the changes in the electronic density that occur when the confined crystallites melt into an amorphous state within the interior of the SBA-15 pores. All of these results point out the main conclusion, which is that the intensity derivative is only a little sensitive to the main phase transitions, while significantly higher changes occur in the zones of secondary endotherms or exotherms, which are connected to the melting or crystallization of the iPP chains confined within the SBA-15 pores. Moreover, the magnitude of the derivative maxima is rather proportional to the real content of mesoporous silica in the composites [60,88]. This verifies that the thorough study of the reflection (100) of SBA-15 particles is a very valuable tool for the characterization of the confinement of polymeric chains that are capable of melting and crystallizing inside their pores.

**Figure 9:**
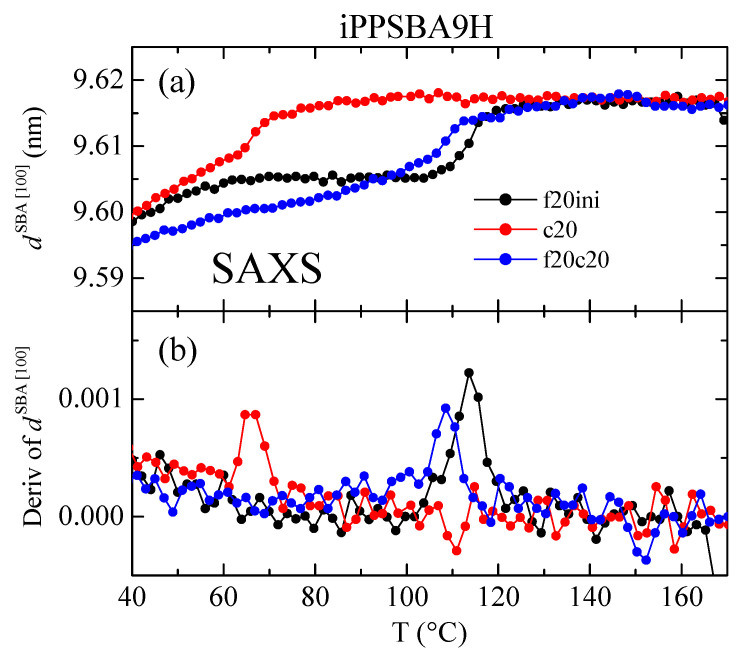
(**a**) Dependence on temperature of the position of the SBA-15 (100) diffraction in the iPPSBA9H nanocomposite relative to the initial heating (f20ini), cooling (c20) and subsequent heating (f20c20), with all of them scanning at 20 °C/min. (**b**) Corresponding derivative of that variation. Black color stands for f20ini, red for c20 and blue for f20c20 in the different plots.

The position of the (100) SBA-15 reflection has been reported to remain almost unaltered in iPP composites prepared via extrusion [67,88]. However, Figure 9 indicates that this premise is not accomplished in the nanocomposites synthesized via in situ polymerization in this study. Although shear forces allow the introduction of some polymeric chains within the SBA-15 channels, it is expected that the number of these macrochains is lower than the number that can be within the mesoporous structure when in situ polymerization is employed, this being mainly true if the SBA-15 particles are also used as a catalyst carrier. The filling of pores is supposed to be higher in the latest. Thus, the position of the (100) peak may differ when a comparison is made between neat SBA-15 and the mesoporous particles loaded with iPP.

Based on the results revealed in Figure 5, Figure 6, Figure 7 and Figure 8, it can be established that the polymerization of propylene takes place in a minor but appreciable amount and is confined within the SBA-15 nanometric pores, although the majority of the macromolecular chains grow outside the silica particles. Once the composite is isolated and processed, the iPP chains are capable of crystallizing into the monoclinic polymorph, since the cooling applied from the melt is fast; this leads to two types of well-differentiated crystallites: firstly, some of a small size, which are those developed in the chains confined within the nanometer spaces of the mesoporous silica; and secondly, crystals that are organized in the macrochains located externally surrounding the silica particles. This great difference in the crystallite size is responsible for the appearance of two separated melting processes: a minor peak at a low temperature that corresponds to the confined crystals, and another at the regular temperature for the melting of iPP chains with specific microstructural characteristics that are provided during the polymerization.

Again, the variations in the position of the SBA-15 peak are very much sensitive to the transitions involving confined iPP crystals. Now, the confined crystallization on cooling is much more clearly observed (compare Figure 7b and Figure 9b).

As a general conclusion, therefore, determining the variation in both the intensity and position of the SBA-15 (100) diffraction is a very appropriate tool for analyzing the iPP chains confined in the SBA channels.

### 2.4. Rheological Response

The incorporation of all types of fillers can affect the viscoelastic behavior, in the molten state, of polymers that are used as matrices. Therefore, the evaluation of the rheological response of (nano)composites is a very important operation, as it helps to guarantee the subsequent processability of the resultant materials. Despite the fact that the synthetic procedure is similar for the two prepared sets of iPP-based composites, by using the SBA-15 particles as a catalyst support, the involvement of two different metallocene catalysts has led to important differences in these materials; these are basically related to changes in their molar mass, their polydispersity and microstructural details (stereo and regio-regularity of chains). These molecular parameters play important roles in the materials’ morphological characteristics, thermal stability, location of phase transitions, features of confinement and crystalline details (some of them analyzed above). Thus, some variation in the rheology of these systems is also expected.

**Figure 10:**
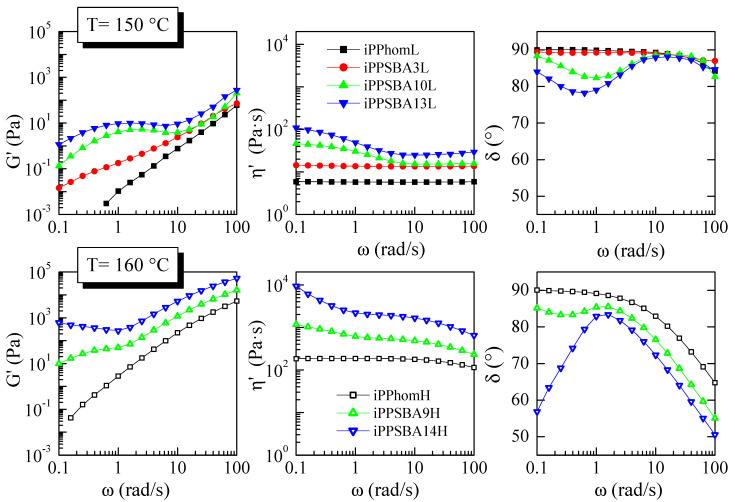
Shear storage modulus (*G*′) (**left** representations), dynamic viscosity (η′) (**middle** representations) and phase angle (δ = tan^−1^ (*G*″/*G*′)) (**right** representations) for some of the materials as a function of frequency; data of iPPhomL and its composites at 150 °C (**upper** plots), and data of iPPhomH and its composites at 160 °C (**lower** plots).

Figure 10 presents the linear viscoelastic data for some of the materials. The shear storage modulus (*G*′), dynamic viscosity (*η*′ = *G*″/ω) and phase angle (δ = tan^−1^ (*G*″/*G*′)) are displayed. The data of the set of iPPhomL materials were obtained at a T^experiment^ of 150 °C, while those of iPPhomH and its composites were obtained at a T^experiment^ of 160 °C. These temperatures were chosen considering the similar value of (T^experiment^ − T_m_) for the two sets of samples, which means that an analogous state is expected in both families.

Homopolymers display viscosities according to their molecular weight. The corresponding zero shear-rate viscosity (η_0_) of iPPhomL has a value of approximately 6 Pa.s, which is approximately 30 times lower than that of iPPhomH. Moreover, iPPhomL shows a Newtonian response in the whole analyzed frequency interval, while iPPhomH displays the beginning of the shear thinning region. At the same time, the storage moduli of both homopolymers display the expected ω^2^ dependency at low frequencies. Assuming that the dynamics of the homopolymers are mainly affected by the size of their macromolecules, their η_0_ and weight-averaged molecular weights should follow a relation of η_0_~*M_w_^a^*, with *a* = 3.4–3.7, which is valid for lineal simple polymers [90]. In the case of iPPhomL and iPPhomH, the values of η_0_ and *M_w_* give *a* = 3.5, which is well in the expected range.

As expected, the dynamic moduli of a given family increase as the SBA-15 content does. This rise is considerable even at low contents, as can be seen when comparing iPPhomL and iPPSBA3L data. The presence of SBA-15 particles and their interactions with the iPP polymer introduce a slow relaxation process (relatively large relaxation time) that has a more noticeable effect on *G*′ than on η′, and mainly at low frequencies [91]. For example, the *G*′ at 0.64 s^−1^ increases approximately 1000 and 40 times when adding 10 and 9 wt.% of SBA-15 to iPPhomL and iPPhomH, respectively; meanwhile, η′ augments just ~6 and 4 times. A large effect is expected at a given filler content in iPPhomL due to its lower molecular weight [91], thus SBA-15 particles having a higher impact as reinforcement.

The dynamic data presented concerning the phase angle emphasize the large effect that the presence of SBA-15 has on *G*′ compared to *G*″ (or η′) in both systems, since δ rapidly decreases with an increasing filler content. The phase angle of the homopolymers reaches 90° in the Newtonian plateau and monotonically decreases with the frequency. As the filler content rises, even with the smaller concentration employed, the curve of δ(ω) acquires an S-shaped form that is emphasized as the SBA-15 concentration increases. In fact, the data begin to reveal a transition that occurs from a liquid to solid-like behavior as the filler concentration increases, mainly in the case of the phase angle at low frequencies The departure from the simple rheological behavior displayed by the homopolymers towards a solid-like behavior (when *G*′ becomes similar or even larger than *G*″ and δ approaches or ends up below 45°) indicates the existence of important interactions between particles. This transition has been described with regard to the development of a transitory network, which is associated with the fractal aggregate percolation of the fillers connected by bridging polymeric chains [92,93]. This has been named rheological percolation. iPPSBA14H is practically in this condition.

The linear viscoelastic response of the two sets of polymers here analyzed is somehow different to that shown by the iPP nanocomposites prepared via extrusion [63]. In this case, a jump from liquid to solid-like behavior was observed in the SBA-15 content range of7–8 to 12–13 wt%. Moreover, the rheological magnitudes observed in the composites comprising up to 7–8 wt.% of filler were very analogous to those exhibited by the homopolymers, with the differences being only very slight. In the materials here studied, the practically percolated structure appears at a rather similar filler concentration, although their responses at the lowest contents of SBA-15 are significantly different. These observations seem to indicate the importance of using this protocol for the preparation of nanocomposites with regard to the chain’s dynamics.

## 3. Materials and Methods

### 3.1. Materials and Chemicals

The SBA-15 particles were purchased from Sigma-Aldrich (specific surface area, S_BET_ = 517 m^2^/g; total pore volume, V_t_ = 0.83 cm^3^/g; average mesopore diameter, D_p_ = 6.25 nm [62]). Prior to its use, the SBA-15 was dried under nitrogen for 2 h at 300 °C. Then, it was cooled and stored under dry nitrogen.

Toluene (VWR Chemicals) was purified via refluxing over metallic sodium under a dry nitrogen atmosphere to avoid the presence of traces of water and oxygen. Both propylene (Praxair 2.5) and nitrogen (Contse) were passed through oxygen-trap columns and molecular sieves before their use.

The catalysts *rac*-ethylenebis(indenyl)zirconium (IV) dichloride (*rac*-Et[Ind]_2_ZrCl_2_) and *rac*-dimethylsilylbis(1-indenyl) zirconium (IV) dichloride (*rac*-Me_2_Si[Ind]_2_ZrCl_2_) (both from Strem), together with the cocatalyst, which was a modified methylaluminoxane (MMAO-12.7 wt. % Al in toluene solution, Aldrich) were used as received. All manipulations were performed under dry nitrogen gas using standard Schlenk techniques.

### 3.2. Preparation of the Supported Catalyst

#### Impregnation of Pre-Activated Zr Catalysts Using MAO on SBA-15 (PA Method)

The immobilization of MMAO pre-activated Zr catalysts on SBA-15 particles (method PA) was the method used to impregnate the catalysts on the mesoporous SBA-15 silica. The pre-activated catalysts were prepared using 2 μmol of zirconocene in a toluene solution and 2.7 mL of MMAO with a Al/Zr ratio of 300. This was stirred for 15 min at room temperature and then the MMAO pre-activated catalyst was mixed with 500 mg of the support in toluene and stirred for 10 min. Then, a small volume (~2 mL) of the clear supernatant liquid was tested in polymerization conditions, with the further addition of MMAO (the same used for the polymerization runs). This polymerization test with this clarified supernatant solution did not lead to any activity, confirming that no catalyst remained in the supernatant solvent [45].

### 3.3. Propylene Polymerizations

Polymerizations were carried out in a 500 mL Büchi glass ecoclave. The reactor was filled with toluene (250 mL), an adequate amount of the MMAO cocatalyst (2.7 mL when using *rac*-Et[Ind]_2_ZrCl_2_ and 4.5 mL when using *rac*-Me_2_Si[Ind]_2_ZrCl_2_) in order to reach a catalyst/cocatalyst ratio of 300, ([Al]/[Zr] = 300), as well as the zirconocene catalyst and propylene. Polymerizations using the *rac*-Et[Ind]_2_ZrCl_2_ catalyst took place at 25 °C, while those employing the *rac*-Me_2_Si[Ind]_2_ZrCl_2_ catalyst were carried out at −5 °C. All of the reactions were performed at a pressure of 1.2 bar for propylene. The preparation of the nanocomposites, using different mesoporous silica contents via in situ polymerization, was performed when the catalyst was immobilized on the SBA-15 particles by employing different reaction times until a given amount of propylene was consumed. Then, the polymerization reactions were stopped by adding 5 mL of ethanol and letting the unreacted propylene out of the reactor. The polymerization mixtures were precipitated via the addition of ethanol (Aroca, 96%) acidified with 10% HCl (VWR, 37%), and the materials were then collected and washed twice using ethanol before drying under vacuum at room temperature. Thus, different materials were obtained and stored.

### 3.4. Characterization of Homopolymers and Nanocomposites

#### 3.4.1. Size Exclusion Chromatography

The molecular weights of some of the synthesized materials were evaluated using size exclusion chromatography (SEC) at 145 °C in Waters GPC/V 2000 system equipped with both refractive index and viscosimeter detectors. A set of three columns of the PL Gel type was used, with 1,2,4-trichlorobenzene employed as a solvent, and a flow rate of 1 mL/min. The analyses were calibrated using the polystyrene standards of narrow molecular mass distributions. The molecular weights and polydispersity index (M_w_/M_n_) obtained are listed in Table 1. The removal of silica is necessary in order to perform this type of experiment. This was carried out as follows: a solution of methanol and 2 *v*.% of hydrofluoric acid was prepared at room temperature, with the required amount of each composite being dispersed. Then, the dispersion was stirred overnight at room temperature. Later, it was filtered, washed thoroughly three times with methanol and dried under vacuum until constant weight was obtained. Finally, a thermogravimetric test was carried out to verify that the silica had been completely removed.

#### 3.4.2. Nuclear Magnetic Resonance

The tacticity was determined via carbon nuclear magnetic resonance analysis, ^13^C NMR, using polymeric solutions prepared in 1,1,2,2-tetrachloroethane-*d*_4_ (70 mg/1 mL) at 100 °C and a Bruker Avance III/500 spectrometer operating at 125.76 MHz. A minimum of 4000 scans were recorded using broadband proton decoupling, with an acquisition time of 1.3 s and a pulse delay of 5 s. The removal of silica is necessary in order to perform this type of experiment. This was carried out as was just mentioned for the SEC measurements.

#### 3.4.3. Preparation of Films

The powders obtained after polymerization were processed as films via compression molding in a Collin P-200-P press between hot plates at 190 °C for 3 min without pressure, followed by 3 min at a pressure of 30 bar. A further fast-cooling process was applied using cold water for 3 min at 30 bar. These films were named as follows: iPPhomL or iPPhomH for the iPP homopolymers synthesized under homogeneous conditions, with *L* or *H* indicating their low or high molecular weight depending on the catalyst used; and iPPSBA*x*L or iPPSBA*y*H for the iPP composites containing mesoporous SBA-15, with x or *y* being the average SBA-15 wt. % content estimated via TGA, and *L* or *H* referring to their low or high molecular weight, respectively.

#### 3.4.4. Thermogravimetric Analysis

Dynamic thermal gravimetric experiments (TGA) were performed from 40 °C up to 700 °C in the Q500 equipment of TA Instruments under an air or nitrogen atmosphere (90 mL·min^−1^) at a heating rate of 10 °C/min. The samples were discs cut from the films with a diameter of4 mm, leading to a sample weight of approximately 4 mg. These measurements were carried out for assessment of the final SBA-15 content in a given nanocomposite, as well as for the evaluation of the degradation processes of the resulting materials.

#### 3.4.5. Scanning Electron Microscopy

Scanning electron microscopy, SEM, was carried out at room temperature in a XL30 ESEM Philips instrument for analyzing the fracture surface found in the films of different composites. The samples were coated with a layer of 80:20 Au/Pd alloy and deposited in a holder prior to observation.

#### 3.4.6. Differential Scanning Calorimetry Analysis

Calorimetric analyses were carried out in a TA Instruments Q100 calorimeter connected to a cooling system and calibrated using different standards. The sample weights were approximately 3 mg for all of them. A temperature interval of −40 to 180 °C was studied at a heating rate of 20 °C/min. For the determination of the crystallinity, a value of 160 J/g was used as the enthalpy of fusion of a perfectly crystalline material [79,94,95].

#### 3.4.7. X-ray Experiments with Conventional and Synchrotron Radiation

Wide-angle X-ray diffraction (WAXD) patterns at room temperature, in the reflection mode, were recorded in order to examine the crystalline structure of the polymeric matrix by using a Bruker D8 Advance diffractometer equipped with a PSD Vantec detector (from Bruker, Madison, WI, USA). Cu Kα radiation (λ = 0.15418 nm) was used, operating at 40 kV and 40 mA. The parallel beam optics were adjusted using a parabolic Göbel mirror with a horizontal grazing incidence Soller slit of 0.12° and a LiF monochromator. The equipment was calibrated using different standards. A step scanning mode was employed for the detector. The diffraction scans were collected with a 2θ step of 0.024° and 0.2 s per step.

Real-time variable-temperature simultaneous SAXS/WAXS experiments were carried out using synchrotron radiation in the beamline BL11-NCD-SWEET at ALBA (Cerdanyola del Vallès, Barcelona, Spain) at a fixed wavelength of 0.1 nm; these were performed in order to analyze the hexagonal ordered arrangement of the mesoporous SBA-15 silica and the crystalline details of the polymer component. An ADSC detector was used for SAXS (off beam, at a distance of 292 cm from sample) and a Rayonix detector was used for WAXD (at approximately 14 cm from sample, and a tilt angle of approximately 30 degrees). A Linkam Unit was employed for the temperature control. The calibration of spacings was obtained by using silver behenate and Cr_2_O_3_ standards. The initial 2D X-ray images were converted into 1D diffractograms, as a function of the inverse scattering vector, *s* = 1/*d* = 2 sin θ/λ. Film samples of approximately 5 × 5 × 0.1 mm were employed in the synchrotron analysis.

#### 3.4.8. Rheological Response

Rheological characterization was carried out in the small-amplitude oscillatory shear mode using a dynamic rotational rheometer TA Instruments ARG2 (New Castle, DE, USA). The tests were performed under a nitrogen atmosphere and using parallel plates that were 25 mm in diameter, a frequency range of 0.1 to 100 rad/s, and a temperature interval of 150–200 °C. All tests were carried out at small stresses in order to assure the linearity of the dynamic responses [90].

## 4. Conclusions

In this study, SBA-15 microparticles were used as support for two different metallocene catalysts in order to prepare new nanocomposites based on commodity polypropylene via in situ polymerization. These hybrid particles, achieved using the pre-activation approach, enabled two families of materials with very different molar masses and distinct contents with regard to stereodefects to be obtained. The mesoporous silicas were, firstly, functionalized using the catalytic system (metallocene catalyst and cocatalyst) for their role as a carrier in the polymerization stage, although their maintenance in the final material allowed them to also act as reinforcement. The protocol used to impregnate the catalytic system involved two stages: (a) the contact of the cocatalyst and each zirconocene used, and (b) the immobilization of the catalytic complex onto the SBA-15 particles. The average activity achieved in the set of materials prepared by using the *rac*-Et[Ind]_2_ZrCl_2_ catalyst was higher than that in the composites obtained using the Me_2_Si[Ind]_2_ZrCl_2_ catalyst; this is because the first zirconocene was less sensitive to impurities. The distribution of the SBA-15 silica in the resultant polypropylene-based materials seemed to be rather uniform, and large-sized agglomerates of inorganic particles were not noticeable.

A secondary endothermic process was observed at approximately 105 °C in all of the nanocomposites, independent of their microstructural characteristics, indicating the development of thin crystals. In addition, it was found that the main melting was located at approximately 136 °C for the set of samples prepared using the Et[Ind]_2_ZrCl_2_ zirconocene and at approximately 148 °C for those synthesized using the Me_2_Si[Ind]_2_ZrCl_2_ catalyst. The secondary endotherm was ascribed to the melting of those crystals confined inside the silica channels. This was confirmed by analyzing the intensity of the main (100) SBA-15 diffraction in the SAXS region, which, importantly, varied in a temperature interval that correlated very well with the DSC results. A very good agreement with these DSC curves was also found using the derivative of the main SBA-15 reflection position, since the iPP chains grew during polymerization either within the silica pores or on the outer surface of the SBA-15 particles, with the former altering the location of the SBA-15 primary diffraction.

The reinforcing role of SBA-15 can be clearly deduced from the rheological response of all these materials, with the values of the shear storage modulus and viscosity increasing with the incorporation of this mesoporous silica when compared to those from the neat iPP matrices. This increase becomes higher as the SBA-15 content is enlarged. Moreover, rheological percolation is reached and a liquid to solid-like behavioral transition is noted in the terminal zone for the composites created using a quantity of SBA-15 that is higher than 3 wt.%, independent of the molecular mass of the corresponding iPP matrix.

## Figures and Tables

**Table 1:** Reaction time and average activity during polymerizations, together with the molecular weight and polydispersity of neat polypropylenes synthesized under homogenous conditions and of iPP/SBA nanocomposites ^a^ prepared in situ using a PA heterogenization protocol in order to support catalysts ^b^. Average values of the content of mesoporous SBA-15 are also given in the last column.

Sample	Reaction Time (min)	Average Activity (Kg·mol^−1^_Zr_·h^−1^)	M_w_ (g/mol)	M_w_/M_n_	SBA-15 Content (wt.%)
iPPhomL	10	13,500	42,000	2.50	0
iPPSBA3L	137	3300	49,600	1.82	3.2
iPPSBA6L	10	14,200	45,700	1.88	6.1
iPPSBA10L	20	4400			9.9
iPPSBA13L	11	8200	43,400	1.91	12.5
iPPSBA82L	1	15,900	-	-	82.0
iPPhomH	60	780	112,100	1.94	0
iPPSBA9H	100	990	168,531	4.42	9.0
iPPSBA14H	120	900	162,343	4.81	14.0
iPPSBA23H	30	1800	157,908	4.45	23.4
iPPSBA29H	140	300	-	-	28.9

^a^ The iPPhomL or iPPhomH are the homopolymers, and L or H indicate low or high molar masses. iPPSBA*x*L or iPPSBA*y*H are the iPP composites, *x* or *y* are the SBA-15 wt. % content, and L or H correspond again to low or high molar masses, respectively. ^b^ P_propylene_ = 1.2 bar; catalyst loaded in the support = 2 μmol/g; impregnation time = 10 min; T = 25 °C for the *rac*-Et[Ind]_2_ZrCl_2_ catalyst and −5 °C for the *rac*-Me_2_Si[Ind]_2_ZrCl_2_ one.

**Table 2:** Average SBA-15 content (determined using TGA experiments performed under inert and air conditions), and the relative content of propylene tactic sequences at the pentad level for *mmmm* and at the triad level for *mm*, *mr*, *rr* and for the regio-defects.

Sample	SBA-15 Content (wt.%)	*mmmm* (mol%)	*mm* (mol%)	*mr* (mol%)	*rr* (mol%)	Regio-Defects (mol%)
iPPhomL	0	78.6	87.0	8.5	4.4	1.7
iPPSBA3L	3.2	81.1	89.2	7.7	3.1	1.3
iPPSBA6L	6.1	79.8	88.4	8.1	3.4	0.8
iPPSBA10L	9.9	77.9	87.1	9.1	3.8	0.8
iPPSBA13L	12.5	81.3	89.2	7.4	3.4	1.5
iPPSBA82L	82.0	-	-	-	-	-
iPPhomH	0	82.7	89.5	6.7	3.1	0.6
iPPSBA9H	9.0	85.4	91.1	5.6	2.5	0.8
iPPSBA14H	14.0	88.1	93.8	4.5	1.7	0.0
iPPSBA23H	23.4	83.3	90.1	5.9	3.3	0.6
iPPSBA29H	28.9	-	-	-	-	-

**Table 3:** Average SBA-15 wt.% content calculated using TGA measurements; the enthalpy and its crystallinity (normalized to the actual iPP content in the material) are estimated using DSC for the first melting and crystallization (ΔH_m_^NORM^, ΔH_C_^NORM^, f_c_^NORM^_m_ and f_c_^NORM^_C_, respectively) together with the melting (T_m_) and crystallization (T_c_) temperatures.

Sample	SBA-15 Content (wt.%)	ΔH_m_^NORM^ (J/g)	f_c_^NORM^_m_ (%)	T_m_ (°C)	ΔH_C_^NORM^ (J/g)	f_c_^NORM^_C_ (%)	T_c_ (°C)
iPPhomL	0	90	56	135.5	85	53	102.5
iPPSBA3L	3.2	93	58	136.0	90	56	103.0
iPPSBA6L	6.1	96	60	136.0	87	55	104.5
iPPSBA10L	9.9	103	64	136.5	91	57	105.0
iPPSBA13L	12.5	100	62	136.5	91	57	105.0
iPPhomH	0	93	58	148.0	96	60	113.0
iPPSBA9H	9.0	92	58	148.5	88	55	112.0
iPPSBA14H	14.0	90	56	148.5	85	53	112.5
iPPSBA23H	23.4	86	55	148.5	80	50	114.0
iPPSBA29H	28.9	92	58	148.5	78	49	114.0

## Data Availability

Data are contained within the article.

## Sample Availability

Samples are available from the authors.
